# Role of responsive neurostimulation and immunotherapy in refractory epilepsy due to autoimmune encephalitis: A case report

**DOI:** 10.3389/fneur.2022.1028290

**Published:** 2022-11-02

**Authors:** Stephanie H. Chen, Pamela K. O'Dea, Bahareh Sianati, David R. Benavides

**Affiliations:** ^1^Department of Neurology, University of Maryland School of Medicine, Baltimore, MD, United States; ^2^Department of Neurology, Indiana University School of Medicine, Indianapolis, IN, United States

**Keywords:** autoimmune-associated epilepsy, RNS, refractory epilepsy, immunotherapy, autoimmune encephalitis

## Abstract

Autoimmune encephalitis (AE) frequently presents with seizures in the acute setting. Seizures are often refractory to anti-seizure medications (ASM) but have been shown to be responsive to immunomodulatory therapies. A subset of patients with AE continues to have refractory epilepsy, recently named “autoimmune-associated epilepsy (AAE),” for years after the acute AE presentation. Optimal treatment for AAE has not been determined. Furthermore, the efficacy of neuromodulation and immunotherapy has not been well established in AAE. Here, we report a patient with probable autoantibody negative AE who initially presented with new onset refractory status epilepticus (NORSE). After his acute presentation, he continued to have frequent seizures that were refractory to four ASMs at therapeutic doses. A responsive neurostimulation (RNS^®^, NeuroPace) system was implanted for diagnostic and therapeutic purposes, with minimal change in seizure frequency. Due to continued frequent seizures despite ASMs and neurostimulation, he underwent a trial of immunotherapy consisting of high-dose intravenous (IV) corticosteroids and intravenous immunoglobulin (IVIG). Despite the addition of immunotherapy to his treatment regimen, the patient experienced no significant clinical or electrographic change in seizure frequency. This case does not support the use of immunotherapy for treatment of AAE and illustrates the need for consensus guidelines in the management of patients with AAE. Further, the use of electrocorticography (ECoG) data provided an objective surrogate measure of seizure frequency; this may support the role for early neuromodulation in the management of AAE.

## Introduction

Seizures and status epilepticus (SE) are common complications of autoimmune encephalitis (AE) (1) and are likely due to inflammatory processes in the brain. Retrospective analysis of cases of autoimmune status epilepticus have shown that the disorder is associated with high mortality and significant risk for major neurologic disability (2). However, refractory epilepsy is a rare complication of AE and little is known about the pathophysiology of autoimmune-associated epilepsy (AAE). It is postulated that AAE is caused by ongoing inflammatory processes or the damage caused to the neuronal network by an initial inflammatory insult (3). Recent case series have discussed adjunctive therapy in treatment-refractory AAE (4, 5). However, consensus guidelines in the management of AAE remain undefined.

In this report, we provide a detailed account of the clinical and laboratory findings of a young man with AE complicated by AAE refractory to multiple anti-seizure medications (ASMs). We review ASM management, surgical evaluation, and neuromodulation therapy with a responsive neurostimulation (RNS^®^, NeuroPace) system. Finally, we report the clinical and RNS-reported effect of immunotherapy with high-dose intravenous (IV) corticosteroids and intravenous immunoglobulin (IVIG) on seizure frequency and severity. This case does not support the use of immunotherapy for treatment of chronic refractory epilepsy due to AE. This case illustrates the need for consensus guidelines in the management of patients with AAE. It also raises the question of the role of early neurostimulation as a diagnostic and therapeutic option in AAE.

## Case presentation

A 28–year-old man with a >2-year history of focal impaired awareness seizures and generalized tonic-clonic (GTC) seizures was referred for evaluation at our National Association of Epilepsy Center (NAEC) Level IV Epilepsy Center. He had a first-time seizure at age 26 (27 months prior to referral) at work, resulting in a fall. Three days later, he experienced two additional falls at work with tongue bite and associated shaking. EMS was contacted for transfer to a local emergency department (ED) for evaluation. Basic serum tests, CT head, and electroencephalogram (EEG) were unremarkable. He was at his baseline, albeit amnestic to the initial event, and was discharged home. Later at home the same evening, he had another GTC seizure lasting 5 min. EMS transferred him to another local hospital, where CT head was repeated and normal. While in the ED, he had another GTC seizure, which resolved with IV lorazepam. Rapid MRI brain was performed and unrevealing for any structural abnormalities; stat EEG showed generalized slowing. He was noted to have a prolonged postictal period without returning to baseline and was transferred to the ICU for close monitoring. While in the ICU, he had a fourth GTC seizure within 24 h; he was treated with phenytoin and transferred to a tertiary care center for management of status epilepticus.

**Figure 1:**
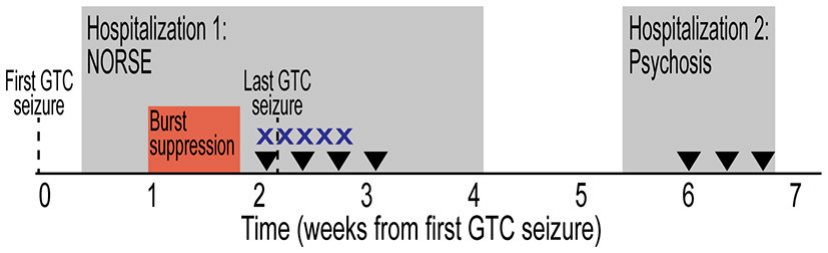
Initial clinical presentation timeline. Timeline depicted from first GTC seizure. Hospitalizations designated by gray rectangles. Period of burst suppression designated by red box. Treatment with high-dose intravenous corticosteroids (X) and plasmapheresis (inverted triangle) are indicated. GTC, generalized tonic-clonic; NORSE, new onset refractory status epilepticus.

He had a prolonged hospitalization at the tertiary care center (Figure 1), totaling 26 days undergoing extensive evaluation and management for new-onset refractory status epilepticus (NORSE) (6). Upon arrival, continuous video EEG (cvEEG) monitoring was initiated, and he was found to have left temporal lateralized periodic discharges (LPDs) and frequent electrographic seizures. His ASM regimen included a phenytoin load and therapeutic doses of levetiracetam and lacosamide. Left temporal lobe seizures continued to cluster every 30–40 min. Maintenance phenytoin was increased and standing clonazepam was added three times daily. Ketamine was eventually added for refractory seizures. Electrographic seizures continued, he was intubated for airway protection, and additional anesthetic agents were added including IV infusions of midazolam, fentanyl, and high-dose propofol.

The differential diagnosis was broad, including infectious, autoimmune, toxic/metabolic, and neoplastic or paraneoplastic etiologies. He had no known viral or infectious prodrome, and no fever or flu-like illness. Comprehensive serum and cerebrospinal fluid (CSF) analyses were performed (Table 1). Initial MRI brain showed no structural abnormalities to explain the etiology of his seizures. Subsequent MRI brain imaging 5 days later showed interval rounded configuration of left amygdala/hippocampus with increased T2 hyperintensity suggesting post-ictal edema, with no associated post-contrast enhancement. Upon extubation and weaning of high dose propofol and fentanyl, clinical seizures recurred. High-dose topiramate was added to his ASM regimen. Additionally, given the working diagnosis of possible AE (7), he received 5 days of high-dose IV corticosteroids (1 g methylprednisolone daily) and four sessions of plasmapheresis with no seizure recurrence. Midazolam was subsequently successfully weaned off; phenytoin was also discontinued. Evaluation for occult malignancy with CT chest/abdomen/pelvis and testicular ultrasound were unremarkable. He was discharged on multiple ASMs and no maintenance immunotherapy. Discharge ASM regimen included clobazam 20 mg twice daily, levetiracetam 2,000 mg twice daily, lacosamide 200 mg twice daily, valproic acid 750 mg twice daily, and topiramate 200 mg three times daily.

**Table 1:** Diagnostic investigations.

	**Routine tests**	**Autoimmune tests**	**Results**
**Serologies**	HIV1/2, HSV, EBV, VZV, VDRL, Lyme, Ehrlichiosis, Enterovirus, RMSF Ab panel, Toxoplasma IgM and IgG, West Nile Virus Ab, HHV6 Ab, Blood culture, AFP, Quantiferon Gold	ANA, dsDNA, Ro/SSA, La/SSB, Smith, RNP, anti-TPO Ab, anti-TG Ab, Mayo PAVAL panel (Amphiphysin, AGNA-1, ANNA-1, ANNA-2, ANNA-3, CRMP-5, VGKC, VGCC, PCA-1, PCA-2, PCA-Tr), Athena NeoEncephalitis panel (Hu, CV2, MaTa, VGKC, Amphiphysin, NR1, GAD65, LGI1, CASPR2)	All negative, with exception of low titer TPO and TG Abs
**Urine analyses**	Legionella antigen Histoplasma antigen		All negative
**CSF analyses**	HSV, VZV, Enterovirus, Arbovirus Ab panel (Western Equine, Eastern Equine, St. Louis, LaCrosse), West Nile Virus Ab, Ehrlichia, Lyme, VDRL		All negative
**CSF 1:** *Day 4*	WBC 38 cell/mm^3^ (97% lymphs) RBC 91 cell/mm^3^ Protein 36 mg/dl Glucose 69 mg/dl		
**CSF 2:** *Day 9*	WBC 4 cell/mm^3^ (87% lymphs) RBC 91 cell/mm^3^ Protein 46 mg/dl Glucose 58 mg/dl	NMDAR	All negative
**CSF 3:** *Day 14*	WBC 14 cell/mm^3^ (53% lymphs) RBC 37 cell/mm^3^ Protein 41 mg/dl Glucose 73 mg/dl	Mayo PAC1 panel (Amphiphysin, AGNA-1, ANNA-1, ANNA-2, ANNA-3, CRMP-5, PCA-1, PCA-2, PCA-Tr), GABA_B_R, MaTa, GAD65	All negative
**CSF 4:** *Month 29*	WBC 6 cell/mm^3^ (87% lymphs) RBC 1 cell/mm^3^ Protein 37 mg/dl Glucose 83 mg/dl	Mayo PAC1 panel (Amphiphysin, AGNA-1, ANNA-1, ANNA-2, ANNA-3, CRMP-5, PCA-1, PCA-2, PCA-Tr), NMDAR, GAD65	All negative

HIV, human immunodeficiency virus; HSV, herpes simplex virus; EBV, Epstein Barr virus; VZV, Varicella Zoster virus; VDRL, Venereal disease research laboratory test; RMSF, rocky mountain spotted fever; Ab, antibody; HHV6, human herpes virus 6; AFP, alpha-fetoprotein; ANA, antinuclear antibody; dsDNA, double-stranded DNA; RNP, ribonucleoprotein; TPO, thyroid peroxidase; TG, thyroglobulin; AGNA, anti-glial nuclear antibody; ANNA-1, antineuronal nuclear antibody-1 (Hu); ANNA-1, antineuronal nuclear antibody-2 (Ri); CRMP-5, collapsing response mediator protein 5 (CV2); VGKC, voltage-gated potassium channel; VGCC, P/Q-type voltage-gated calcium channel; PCA-1, Purkinje cell cytoplasmic antibody type 1; PCA-2, Purkinje cell cytoplasmic antibody type 2; PCA-Tr, Purkinje cell cytoplasmic antibody-Tr; NR1, N-methyl-D-aspartate receptor 1 (NMDAR); GAD65, glutamic acid decarboxylase 65-kilodalton isoform; LGI1, Leucine-rich glioma-inactivated protein 1; CASPR2, contactin-associated protein-like 2; CSF, cerebrospinal fluid; lymphs, lymphocytes; GABA_B_R, γ-aminobutyric acid-B receptor.

During his prolonged hospitalization, he developed new onset cognitive deficits, including severely impaired short-term memory, word finding challenges, and visual processing difficulty. He also developed new behavioral problems and personality changes that were out-of-character including labile mood, irritability, impulsivity, and aggression. He reported “war-like hallucinations” that he attributed to levetiracetam. He was initiated on risperidone and melatonin, and his episodes were largely re-directable, necessitating haloperidol only in extreme situations. While there were no GTC seizures, he developed worsening hallucinations, fear, and persecutory delusions. Outpatient medication management, including reducing levetiracetam and adjusting psychoactive medications, failed to control symptoms and the patient was readmitted to the tertiary care hospital (Figure 1). He was treated with plasmapheresis for three sessions and levetiracetam was discontinued with improvement of psychotic symptoms. Upon discharge home, his behavior improved, but he continued to have treatment-refractory epilepsy.

**Figure 2:**
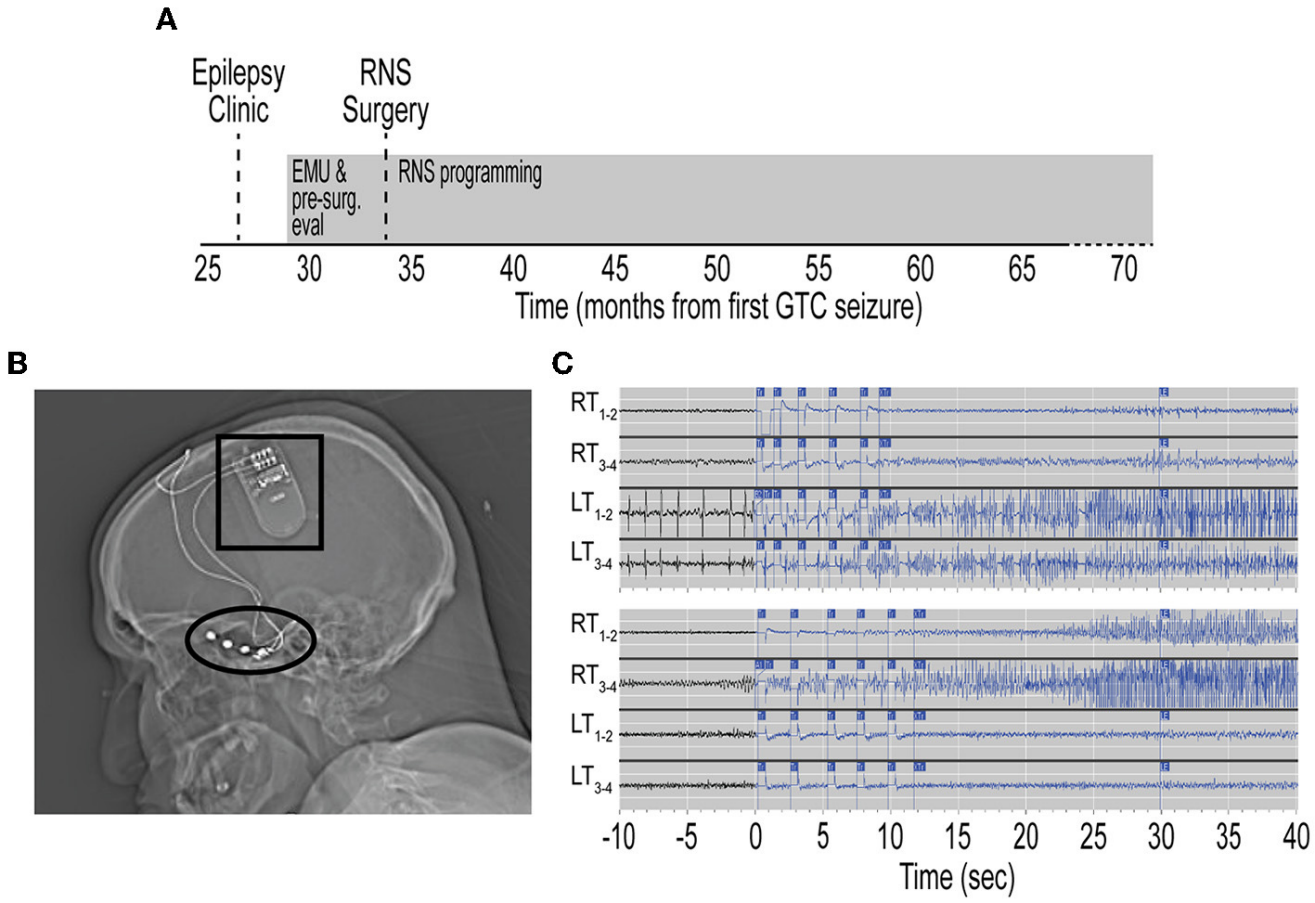
Treatment timeline with RNS placement and temporal lobe seizure capture. **(A)** Timeline from initial referral for evaluation in Epilepsy clinic, pre-surgical evaluation, and RNS surgery. Timeline depicted from first GTC seizure. Evaluations and RNS programming designated by gray rectangles. **(B)** Sagittal head computed tomography scan showing RNS generator (black box) implanted in right frontal skull. Two 4-contact subtemporal lobe strips are implanted bilaterally (black oval). **(C)** Top, representative electrocorticography (ECoG) demonstrating left temporal lobe onset seizures. Time scale represents seconds from RNS seizure detection. Traces show seizure detection in LT1–2 and LT3–4 subtemporal contact. Bottom, representative ECoG demonstrating in right temporal lobe onset seizures. RNS, responsive neurostimulator; EMU, epilepsy monitoring unit; GTC, generalized tonic-clonic; RT, right temporal; LT, left temporal; Tr, treatment.

After 27 months of drug-resistant epilepsy, he presented to our institution for evaluation (Figure 2A). He reported 3–4 seizures/week, poor memory, tremors, and mood instability/irritability. He had three main seizure types, (1) focal aware seizure (FAS), room spinning, feeling of a sudden drop “like on a roller coaster”; (2) focal impaired aware seizure (FIAS), left-sided shaking, labored breathing and foaming at the mouth lasting up to 2 min in duration; and (3) focal to bilateral tonic-clonic seizure (FBTCS), nocturnal convulsions. Additionally, he reported frequent ED visits due to injury, falls, and shoulder dislocations. He was on four ASMs at therapeutic doses, phenytoin 100 mg three times daily, valproate 750 mg three times daily, lacosamide 250 mg twice daily, and clonazepam 2 mg at bedtime. Previously, levetiracetam and topiramate were discontinued due to ineffectiveness and intolerability. His physical examination showed poor memory with 1/3 recall at 5 min, horizontal gaze-evoked nystagmus bilaterally, and an intention tremor, left greater than right, with finger-nose-finger testing. The remainder of the general and neurological examination were unremarkable.

Repeat diagnostics and presurgical epilepsy evaluation were performed at our center including Epilepsy Monitoring Unit admission, where cvEEG demonstrated independent bilateral anterior temporal sharp waves and bilateral independent temporal seizures (Supplementary Figure 1). Repeat CSF analysis showed mild pleocytosis (Table 1). A comprehensive neuropsychological evaluation showed disruption of multiple cognitive domains. MRI brain demonstrated left mesial temporal sclerosis and FDG-PET brain was unremarkable (Supplementary Figure 2). He was presented at our Multidisciplinary Epilepsy Surgery Conference, and the group consensus was to offer bitemporal neurostimulation therapy for seizure reduction. Approximately 34 months after seizure onset, the patient underwent placement of RNS system with two bilateral subtemporal four-contact strips (Figure 2B). Detection settings were honed and accurate in detecting electrographic seizures within 1–2 s of seizure onset (Figure 2C). Stimulation therapy was enabled, and targeted programming was performed on a ~q3month-basis, consistent with the literature (8). He experienced a mild improvement in seizure severity with the addition of RNS therapy. However, there was no significant improvement in seizure frequency, seen on electrocorticography (ECoG) or by patient report.

**Figure 3:**
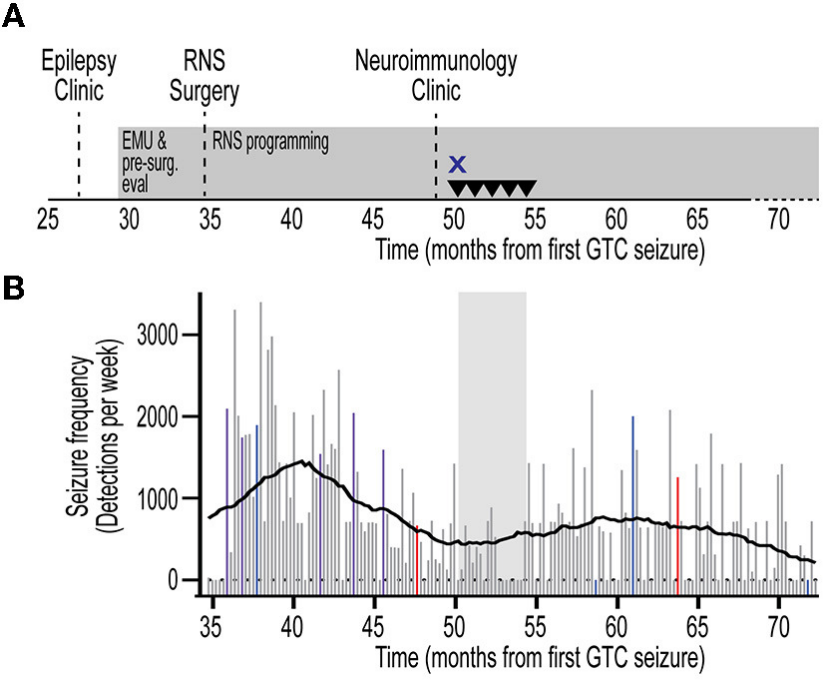
Timeline of immunotherapy and quantitative seizure analysis. **(A)** Timeline from initial referral to evaluation in Epilepsy clinic, pre-surgical evaluation, RNS surgery, and evaluation in Neuroimmunology clinic. Timeline depicted from first GTC seizure. Treatment with high-dose intravenous corticosteroids (X) and intravenous immunoglobulin (inverted triangle) are indicated. RNS programming and treatment continuously employed (gray rectangles). **(B)** Quantification of seizure frequency since RNS placement. Events were extracted from the Patient Data Management System (PDMS) and annotated to yield histogram of seizure frequency in 1-week bins. Missing data are represented as zero events. Data represent detections per week. RNS programming sessions depicted for corresponding week as detection-only change (red), treatment-only change (blue), or detection and treatment changes (violet). Non-programming weeks depicted in gray. Rolling average trendline represents 0^th^ order polynomial of 20 neighbors for each data point to aide in data visualization and reduce visual impact of data fluctuations. Corresponding time of immunotherapy shown (gray shaded area). Note the lack of significant change in seizure frequency during, and following, immunotherapy. RNS, responsive neurostimulator; EMU, epilepsy monitoring unit; GTC, generalized tonic-clonic.

The patient was evaluated in our Neuroimmunology clinic for consideration of immunotherapy (Figure 3A). During this evaluation, historical records were reviewed and the diagnosis of autoantibody-negative but probable AE (7) was confirmed at seizure onset. The APE score was 8 and the RITE score was 10 for this patient (9). Given the history of response to immunotherapy, prominent neuropsychiatric symptoms, persistent CSF pleocytosis (Table 1), and chronic treatment-refractory epilepsy despite multiple ASMs and RNS therapy, he underwent a trial of immunotherapy for the management of AAE. This trial consisted of high-dose IV corticosteroids (1 g methylprednisolone daily for 3 days) and IVIG (2 g/kg in three divided doses) induction, followed by monthly IVIG (0.6 g/kg) for maintenance immunotherapy (Figure 3). He received monthly IVIG treatment and tolerated this immunotherapy treatment regimen well. There was no report of worsening mood symptoms. However, treatment was concluded following four maintenance IVIG doses due to cost barriers. Analysis of objective ECoG data on the Patient Data Management System (PDMS) and patient-reported seizure frequency failed to show a positive effect of immunotherapy on seizure frequency (Figure 3B).

The patient is now 62 months since RNS implantation and continues to have 2–4 FIAS/FBTC seizures per month. He remains on ASM regimen consisting of divalproex 750 mg three times daily, lacosamide 250 mg twice daily, phenytoin 200 mg three times daily, and clonazepam 1 mg twice daily. While he has not seen a significant change in seizure frequency, he has experienced improvement in seizure severity and shortened recovery time postictally. Utilizing the diagnostic capabilities of the RNS device, his seizures have been lateralized, 75% with left temporal onset, 25% with right temporal onset (Figure 2C). He recently completed repeat comprehensive neuropsychological testing and Wada testing, which demonstrated left hemispheric language dominance and that the right hemisphere is independently capable of supporting memory. He is scheduled for a palliative standard left anterior temporal lobectomy (10) with the goal of improved seizure control and quality of life.

## Discussion

Here, we report the clinical course of a patient with autoantibody-negative but probable AE that presented with NORSE. A retrospective chart review was conducted through the electronic medical record assessing clinical data, RNS implantation details, and clinical outcomes. The clinical outcomes included patient-reported seizure severity, frequency, and duration. RNS data were reviewed for long-term ambulatory ECoG. Self-reported clinical monthly seizure frequency and clinical evaluations were compared to ECoG. After years of treatment-refractory epilepsy, he underwent neurostimulation and immunotherapy for AAE. Despite these interventions, his epilepsy remains refractory.

A recent report validated the use of Autoantibody Prevalence in Epilepsy (APE) score and a predictive model using Response to Immunotherapy in Epilepsy (RITE) Score to aid in the management and prognostication in seizures secondary to AE (9). Notably, the APE score was 8 and the RITE score was 10 for this patient. These scores are associated with higher likelihood of harboring specific neural autoantibody, as well as favorable seizure outcome to immunotherapy for symptomatic seizures secondary to AE. Indeed, this patient responded to immunotherapy in the acute management of NORSE. Further, clinical reports have documented that a trial of immunotherapy is justified in the absence of other treatment options (11) in the acute AE setting. However, there is no established role for maintenance immunotherapy in the management of AAE. When offered a trial of immunotherapy for AAE, the patient was apprehensive about the use of corticosteroids given his underlying mood disorder. The use of long-term vs. maintenance immunotherapy as a bridging means after acute seronegative AE and its related refractory epilepsy has not been fully elucidated. On a recent survey, a minority of providers indicated their willingness to use immunosuppressing agents after the first AE attack (12). Additionally, the recurrence rate in seronegative AE remains unknown (13). Therefore, while patients with ongoing neurological disability may be considered for immunotherapy and continued cancer screening (13), any decision regarding immunotherapy should carefully be weighed against side-effect profiles. Our patient tolerated high-dose IV corticosteroids and IVIG without adverse side effects.

The management of AAE remains a significant clinical challenge. The field has been hampered by several factors including mostly retrospective studies, small sample sizes, and clinical and biomarker heterogeneity among reported cohorts. The seronegative AE population is particularly challenging and poorly understood as a group. Estimates on the incidence of AAE have varied widely in the literature, ranging from rare (14–17) to common (18, 19). Several factors have been suggested to predict risk of AAE, including hippocampal structural abnormalities (20), presence of interictal discharges (IED) on EEG (20), immunotherapy delay (19, 20), and seizure severity at onset (18, 19). Further, cell surface antibodies are associated with better outcome, whereas intracellular antigen antibodies, as well as NORSE, have poorer outcomes (3). It has also been posited that more mild clinical syndromes are at higher risk of chronic epilepsy (21). Thus, while clinical patterns are emerging, there are no clear validated predictive models for risk of AAE available to date.

The main therapeutic options for management of AAE are ASMs and immunotherapy (22–25). Adjunctive therapies including neuromodulation have shown safety and efficacy when used in drug-resistant focal epilepsy (26). Recent case-series utilizing neuromodulation in refractory mesial temporal lobe epilepsy in AE with GAD65 antibodies (5) and AAE (4) showed a seizure responder rate in nearly half of patients. Like our patient, even those patients that did not achieve a >50% clinical seizure reduction experienced improvement in seizure severity and duration (4). In this cohort, three of nine patients were reported to have trialed chronic immunotherapy (4). Additionally, the RNS device provided additional information regarding seizure burden and lateralization, ultimately leading to an anterior temporal lobectomy for one patient (5). Despite several available therapeutic options, there is no formally evaluated therapeutic algorithm in AAE.

We report the clinical course of a patient with refractory epilepsy following autoantibody-negative but probable AE without a clear improvement of his clinical course with ASMs, neuromodulation, or immunotherapy. Thirty-four months after acute AE, he underwent implantation of RNS for therapeutic and diagnostic purposes. The ECoGs provided objective insight on electrographic seizure burden and confirmed the lack of meaningful improvement while undergoing immunotherapy. Additionally, the ECoG data lateralized his seizures over several years, facilitating his candidacy for palliative epilepsy surgery.

This case does not support the use of immunotherapy for treatment of chronic refractory epilepsy due to AE. Finally, it illustrates the need for consensus guidelines in the management of patients with AAE. Further studies are needed to determine the role of early neuromodulation in the management of AAE.

## Patient perspective

“Autoimmune encephalitis has messed up my life by not letting me work or do anything fun like I used to do. I used to go out a lot with friends but there's times that I have to pass up on a lot of things because I don't feel safe to do it. The accident happened in [prior state] when I fell and hit my back on a bar. When I moved back to [home state], I was introduced to many doctors that really helped with my problem. The medications that they have me on are lacosamide, divalproex, phenytoin, also clonazepam to help. But when I got the NeuroPace device it helped a lot more than without it. I have had less seizures ever since I had the RNS device installed. It spots the seizures and sends a small shock, which you don't feel at all. With the RNS, you upload your information to a computer that they give you; then your doctor will locate where it happened and add more energy to that strip. I really don't remember much about the IVIG.”

## Data availability statement

The raw data supporting the conclusions of this article will be made available by the authors, without undue reservation.

## Ethics statement

Ethical review and approval was not required for the study on human participants in accordance with the local legislation and institutional requirements. The patients/participants provided their written informed consent to participate in this study. Written informed consent was obtained from the individual(s) for the publication of any potentially identifiable images or data included in this article.

## Author contributions

SC designed and conceptualized study, evaluated patient, collected data, analyzed data, interpreted data, and revised the manuscript. PO'D collected data, analyzed data, and drafted the manuscript. BS analyzed data and edited the manuscript. DB conceptualized study, evaluated patient, analyzed data, and revised the manuscript. All authors contributed to the article and approved the submitted version.

## Funding

This work was supported by the University of Maryland, Baltimore, Institute for Clinical and Translational Research (ICTR) and the National Center for Advancing Translational Sciences (NCATS) Clinical Translational Science Award (CTSA) grant number 1UL1TR003098 (supporting SC) and NIH grant K08 NS114039 to DB.

## Conflict of interest

The authors declare that the research was conducted in the absence of any commercial or financial relationships that could be construed as a potential conflict of interest.

## Publisher's note

All claims expressed in this article are solely those of the authors and do not necessarily represent those of their affiliated organizations, or those of the publisher, the editors and the reviewers. Any product that may be evaluated in this article, or claim that may be made by its manufacturer, is not guaranteed or endorsed by the publisher.

## References

[1] Dalmau J Graus F. Antibody-mediated encephalitis. N Engl J Med. (2018) 378:840–51. 10.1056/NEJMra1708712 29490181

[2] Holzer FJ Seeck M Korff CM. Autoimmunity and inflammation in status epilepticus, from concepts to therapies. Expert Rev Neurother. (2014) 14:1181–202. 10.1586/14737175.2014.956457 25201402

[3] Spatola M Dalmau J. Seizures and risk of epilepsy in autoimmune and other inflammatory encephalitis. Curr Opin Neurol. (2017) 30:345–53. 10.1097/WCO.0000000000000449 28234800 PMC5831325

[4] Chen B Lundstrom BN Crepeau AZ Dacpano L Lopez-Chiriboga AS Tatum WO. Brain responsive neurostimulation device safety and effectiveness in patients with drug-resistant autoimmune-associated epilepsy. Epilepsy Res. (2022) 184:106974. 10.1016/j.eplepsyres.2022.106974 35803065

[5] Feyissa AM Mirro EA Wabulya A Tatum WO Wilmer-Fierro KE Won Shin H. Brain-responsive neurostimulation treatment in patients with GAD65 antibody-associated autoimmune mesial temporal lobe epilepsy. Epilepsia Open. (2020) 5:307–13. 10.1002/epi4.12395 32524057 PMC7278537

[6] Hirsch LJ Gaspard N van Baalen A Nabbout R Demeret S Loddenkemper T. Proposed consensus definitions for new-onset refractory status epilepticus (NORSE), febrile infection-related epilepsy syndrome (FIRES), and related conditions. Epilepsia. (2018) 59:739–44. 10.1111/epi.14016 29399791

[7] Graus F Titulaer MJ Balu R Benseler S Bien CG Cellucci T. A clinical approach to diagnosis of autoimmune encephalitis. Lancet Neurol. (2016) 15:391–404. 10.1016/S1474-4422(15)00401-9 26906964 PMC5066574

[8] Geller EB Skarpaas TL Gross RE Goodman RR Barkley GL Bazil CW. Brain-responsive neurostimulation in patients with medically intractable mesial temporal lobe epilepsy. Epilepsia. (2017) 58:994–1004. 10.1111/epi.13740 28398014

[9] Dubey D Singh J Britton JW Pittock SJ Flanagan EP Lennon VA. Predictive models in the diagnosis and treatment of autoimmune epilepsy. Epilepsia. (2017) 58:1181–9. 10.1111/epi.13797 28555833

[10] Hirsch LJ Mirro EA Salanova V Witt TC Drees CN Brown MG. Mesial temporal resection following long-term ambulatory intracranial EEG monitoring with a direct brain-responsive neurostimulation system. Epilepsia. (2020) 61:408–20. 10.1111/epi.16442 32072621 PMC7154711

[11] Quek AM Britton JW McKeon A So E Lennon VA Shin C. Autoimmune epilepsy, clinical characteristics and response to immunotherapy. Arch Neurol. (2012) 69:582–93. 10.1001/archneurol.2011.2985 22451162 PMC3601373

[12] Abboud H Probasco JC Irani S Ances B Benavides DR Bradshaw M. Autoimmune encephalitis, proposed best practice recommendations for diagnosis and acute management. J Neurol Neurosurg Psychiatry. (2021) 92:757–68. 10.1136/jnnp-2020-325300 33649022 PMC8223680

[13] Abboud H Probasco J Irani SR Ances B Benavides DR Bradshaw M. Autoimmune encephalitis, proposed recommendations for symptomatic and long-term management. J Neurol Neurosurg Psychiatry. (2021) 92:897–907. 10.1136/jnnp-2020-325302 33649021 PMC8292591

[14] Ilyas-Feldmann M Prüß H Holtkamp M. Long-term seizure outcome and antiseizure medication use in autoimmune encephalitis. Seizure. (2021) 86:138–43. 10.1016/j.seizure.2021.02.010 33618141

[15] Wesselingh R Broadley J Buzzard K Tarlinton D Seneviratne U Kyndt C. Prevalence, risk factors, and prognosis of drug-resistant epilepsy in autoimmune encephalitis. Epilepsy Behav. (2022) 132:108729. 10.1016/j.yebeh.2022.108729 35623203

[16] Liu X Guo K Lin J Gong X Li A Zhou D. Long-term seizure outcomes in patients with autoimmune encephalitis, a prospective observational registry study update. Epilepsia. (2022) 63:1812–21. 10.1111/epi.17245 35357695

[17] Yao L Yue W Xunyi W Jianhong W Guoxing Z Zhen H. Clinical features and long-term outcomes of seizures associated with autoimmune encephalitis, a follow-up study in East China. J Clin Neurosci. (2019) 68:73–9. 10.1016/j.jocn.2019.07.049 31331752

[18] Matricardi S Casciato S Bozzetti S Mariotto S Stabile A Freri E. Epileptic phenotypes in autoimmune encephalitis: from acute symptomatic seizures to autoimmune-associated epilepsy. J Neurol Neurosurg Psychiatry. (2022) 93:1194–201. 10.1136/jnnp-2022-329195 35879055

[19] Chen SS Zhang YF Di Q Shi JP Wang LL Lin XJ. Predictors and prognoses of epilepsy after anti-neuronal antibody-positive autoimmune encephalitis. Seizure. (2021) 92:189–94. 10.1016/j.seizure.2021.09.007 34551365

[20] Gifreu A Falip M Sala-Padró J Mongay N Morandeira F Camins Á. Risk of developing epilepsy after autoimmune encephalitis. Brain Sci. (2021) 11:1182. 10.3390/brainsci11091182 34573203 PMC8468512

[21] Casciato S Morano A Fattouch J Fanella M Avorio F Albini M. Factors underlying the development of chronic temporal lobe epilepsy in autoimmune encephalitis. J Neurol Sci. (2019) 396:102–7. 10.1016/j.jns.2018.10.026 30447604

[22] Dalmau J Lancaster E Martinez-Hernandez E Rosenfeld MR Balice-Gordon R. Clinical experience and laboratory investigations in patients with anti-NMDAR encephalitis. Lancet Neurol. (2011) 10:63–74. 10.1016/S1474-4422(10)70253-2 21163445 PMC3158385

[23] Feyissa AM López Chiriboga AS Britton JW. Antiepileptic drug therapy in patients with autoimmune epilepsy. Neurol Neuroimmunol Neuroinflamm. (2017) 4:e353. 10.1212/NXI.0000000000000353 28680914 PMC5489139

[24] Jang Y Kim DW Yang KI Byun JI Seo JG No YJ. Clinical approach to autoimmune epilepsy. J Clin Neurol. (2020) 16:519–29. 10.3988/jcn.2020.16.4.519 33029957 PMC7541993

[25] Toledano M Britton JW McKeon A Shin C Lennon VA Quek AM. Utility of an immunotherapy trial in evaluating patients with presumed autoimmune epilepsy. Neurology. (2014) 82:1578–86. 10.1212/WNL.0000000000000383 24706013 PMC4013813

[26] Morrell MJ RNS System in Epilepsy Study Group. Responsive cortical stimulation for the treatment of medically intractable partial epilepsy. Neurology. (2011) 77:1295–304. 10.1212/WNL.0b013e3182302056 21917777

