# Supplementary figures and images for: Role of responsive neurostimulation and immunotherapy in refractory epilepsy due to autoimmune encephalitis: A case report
Source: Front Neurol. 2022 Nov 2;13:1028290. doi: 10.3389/fneur.2022.1028290 (PMC9666681; doi:10.3389/fneur.2022.1028290)

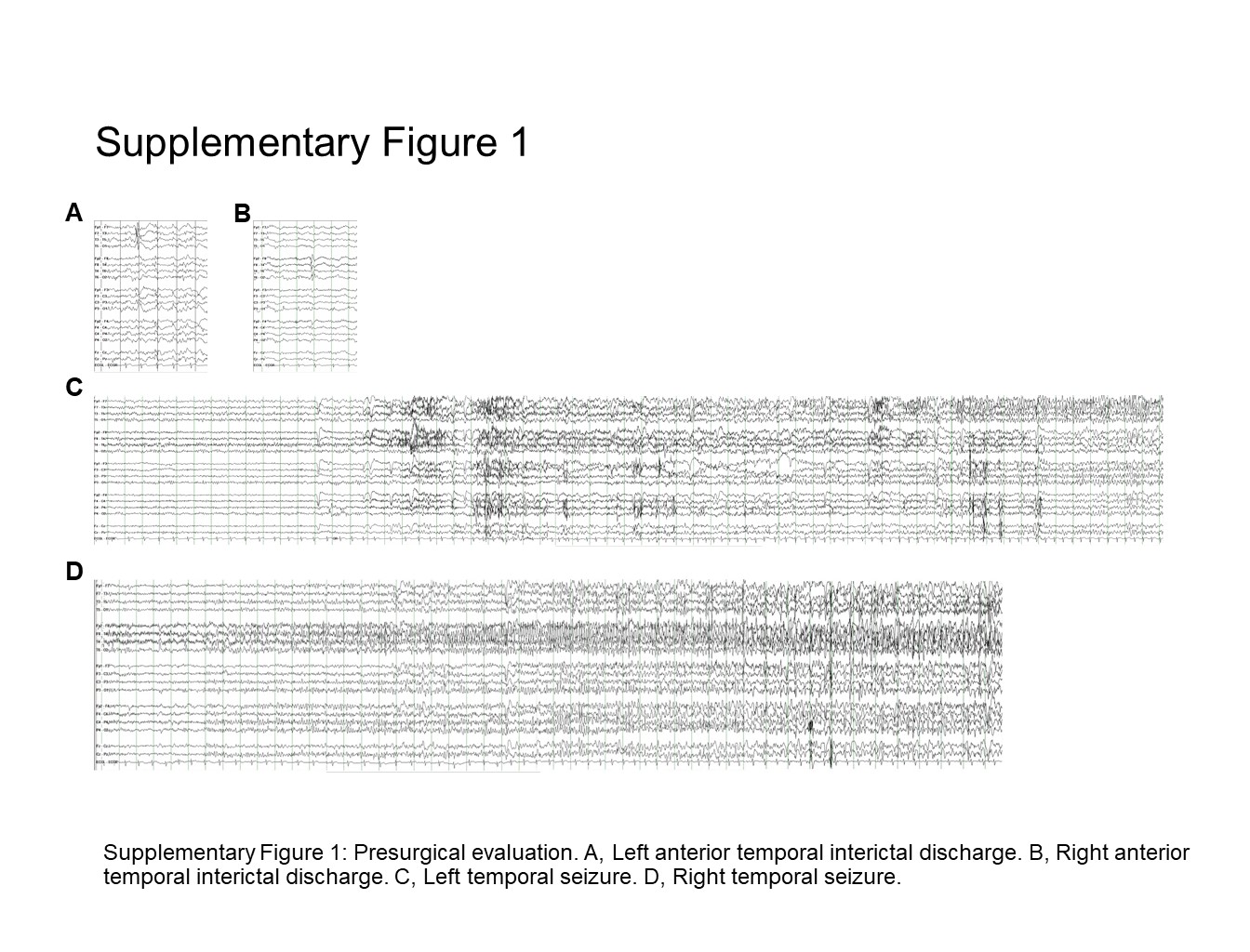

Supplement: Supplementary file 1 [file Image_1.jpg]

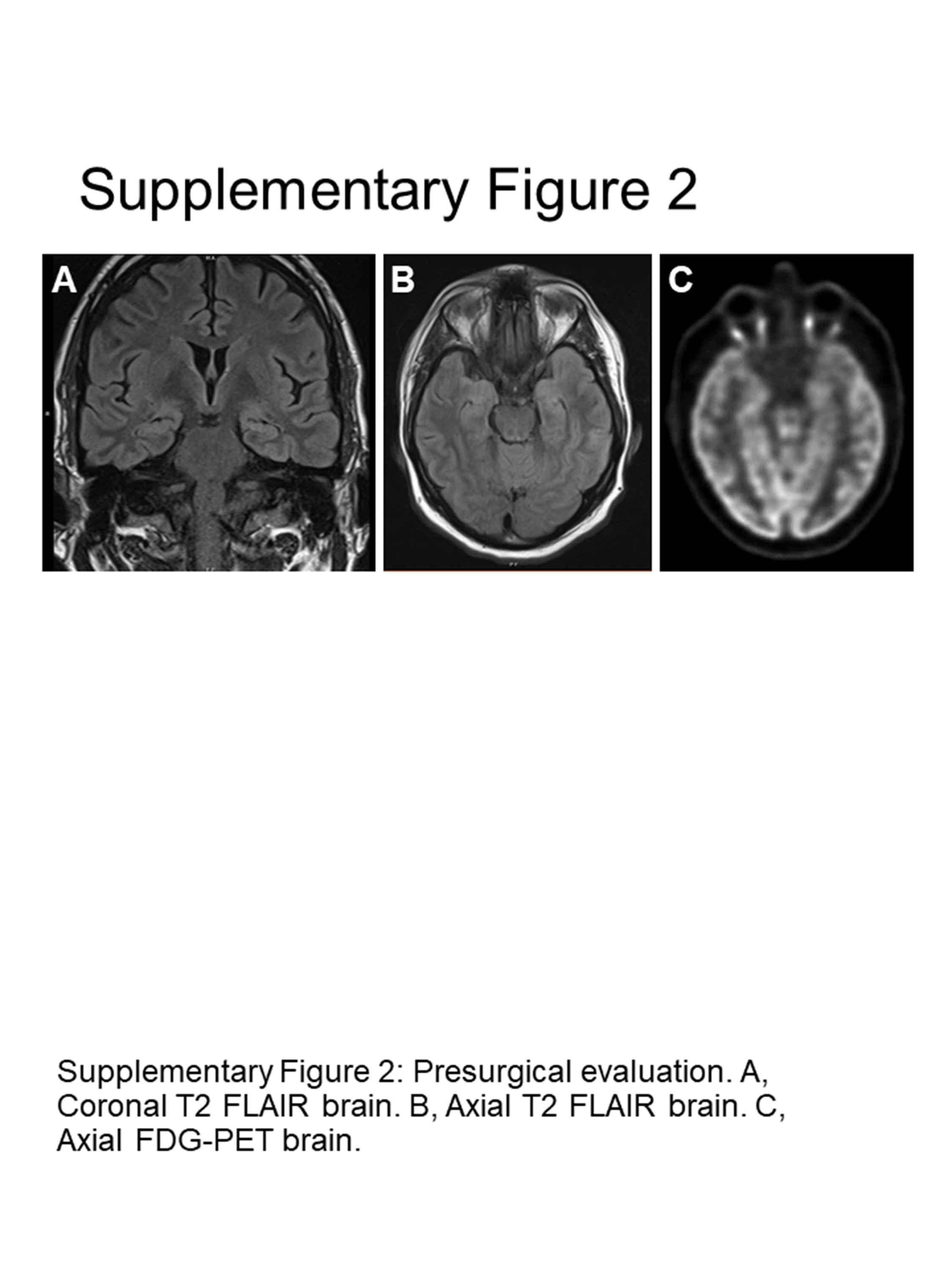

Supplement: Supplementary file 2 [file Image_2.tif]
